# The Properties of Red Blood Cells from Patients Heterozygous for HbS and HbC (HbSC Genotype)

**DOI:** 10.1155/2011/248527

**Published:** 2010-10-13

**Authors:** A. Hannemann, E. Weiss, D. C. Rees, S. Dalibalta, J. C. Ellory, J. S. Gibson

**Affiliations:** ^1^Department of Veterinary Medicine, University of Cambridge, Madingley Road, Cambridge CB3 0ES, UK; ^2^Department of Molecular Haematology, King's College Hospital, London SE5 9RS, UK; ^3^Department of Physiology, Anatomy & Genetics, University of Oxford, Parks Road, Oxford OX1 3PT, UK

## Abstract

Sickle cell disease (SCD) is one of the commonest severe inherited disorders, but specific treatments are lacking and the pathophysiology remains unclear. Affected individuals account for well over 250,000 births yearly, mostly in the Tropics, the USA, and the Caribbean, also in Northern Europe as well. Incidence in the UK amounts to around 12–15,000 individuals and is increasing, with approximately 300 SCD babies born each year as well as with arrival of new immigrants. About two thirds of SCD patients are homozygous HbSS individuals. Patients heterozygous for HbS and HbC (HbSC) constitute about a third of SCD cases, making this the second most common form of SCD, with approximately 80,000 births per year worldwide. Disease in these patients shows differences from that in homozygous HbSS individuals. Their red blood cells (RBCs), containing approximately equal amounts of HbS and HbC, are also likely to show differences in properties which may contribute to disease outcome. Nevertheless, little is known about the behaviour of RBCs from HbSC heterozygotes. This paper reviews what is known about SCD in HbSC individuals and will compare the properties of their RBCs with those from homozygous HbSS patients. Important areas of similarity and potential differences will be emphasised.

## 1. Introduction

Like homozygous HbSS individuals, individuals heterozygous for HbS and HbC (HbSCs) suffer from sickle cell disease (SCD) [1–6]. The condition in HbSC patients (here called HbSC disease cf. HbSS disease in homozygotes) not only has some overlap with that seen in HbSS patients, but also has distinctive laboratory and clinical features identifying it as a separate entity [6–8]. Although HbSC disease is one of the commonest significant genetic diseases worldwide, it is comparatively neglected with very few laboratory or clinical studies addressing the condition directly. Thus, whilst extensive research has been carried out on understanding SCD in HbSS patients, little relates specifically to the pathogenesis in HbSC patients. In clinical trials of potential novel therapies for SCD, HbSC patients are often specifically excluded. Furthermore, most clinical and laboratory features of HbSC disease have been inferred from studies of HbSS, which may not be appropriate. This paper addresses the pathophysiological differences shown by SCD in HbSS and HbSC patients and the diversity in their clinical complications. Particular reference is paid to the transport abnormalities of the RBC membrane. 

## 2. Genotypic Variants of SCD

All SCD patients have the abnormal haemoglobin HbS in their red cells instead of the normal adult HbA [9–12]. HbS results from a single base mutation in codon 6 of the *β*-globin gene which causes a single amino acid substitution in position *β*6 (glutamic acid → valine, with net loss of one negative charge). Homozygous HbSS patients have two copies of the altered gene. The mutation arose in West Africa, where the high prevalence of HbS appears to be due to selection pressure conferred by a relative resistance to malaria. Malaria resistance has also increased the prevalence of a second abnormal Hb, HbC, which like HbS represents one of the most prevalent forms of abnormal human Hb. HbC also has a single mutation/amino acid change at the same position in *β*-Hb, but with lysine replacing glutamic acid (hence net loss of two negative charges). These changes in protein charge may alter how the different Hbs interact and modulate transporter function at the RBC membrane [13]. The charge differences are also used for electrophoretic tests for abnormal Hb, although care must be exercised to exclude certain non-SCD haemoglobinopathies which may mimic HbSC. Homozygous HbCC individuals show few disease symptoms apart from a mild haemolytic anaemia [6]. Heterozygotes of HbA with either HbS or HbC are also largely asymptomatic. Coinheritance of HbS and HbC to produce HbSC heterozygotes, however, results in a clinically significant disease similar, but not identical, to that in HbSS individuals [6–8]. Although globally HbSC heterozygotes represent about a third of SCD cases, their distribution is by no means uniform. HbC appears to have originated in Burkina Faso [6] where HbSC cases may outnumber those of HbSS. In other areas, such as the Middle East and India, HbSC cases are rare. In this context, it is worth pointing out that estimates of the frequency of different haemoglobinopathies are likely to be inexact, relying on outdated or incorrect information [14].

## 3. HbSC Disease as a Unique Clinical Entity

All cases of SCD, including those of HbSC disease, are characterised by shortened red cell life span and chronic anaemia, together with recurrent episodes of more acute vaso-occlusion, tissue ischaemia, and increased mortality [12]. Affected individuals have a poor quality of life with numerous complications, for example, pain, cerebrovascular disease (strokes), renal and pulmonary damage, leg ulcers, and priapism [2]. An important feature of SCD is that the clinical scenario is notably heterogeneous—patients may present with mild forms of the disease which rarely require medical intervention or alternatively with more severe complications warranting frequent hospitalisation and aggressive management. Presumably modifier genes and/or environmental factors are significant, but although this area is now receiving considerable attention, it remains poorly understood at present [15–17]. 

In most cases, HbSC disease is clinically milder than HbSS disease and the various complications of SCD usually occur less often or later in life [8]. For example, leg ulcers and other chronic vascular manifestations occur infrequently. Loss of splenic function is relatively delayed, preserving red cell scavenging and thereby possibly affecting disease complications. Nevertheless, HbSC disease still has a significant impact on patients who show haemolytic anaemia, organ failures (stroke, renal failure, chronic lung disease.), and increased mortality (with a median survival of 60 years for males in USA) [15, 18]. Pregnant women sometimes develop complications having been hitherto asymptomatic [19]. Complications also occur in children with the risk of stroke in childhood being about 100 times greater than that in the general population [18]. Furthermore, in HbSC heterozygotes, some of the serious complications of SCD (such as osteonecrosis) are as common as for HbSS patients and some (e.g., proliferative sickle retinopathy and possibly acute chest syndrome) occur more frequently [8]. This is also apparent for some central auditory and vestibular problems [19].

 Additionally, HbSC is haematologically distinct from HbSS, with higher Hb levels (but lower levels of HbF), lower rates of haemolysis and lower white cell counts [8]. Some of these features are well illustrated in clinical and haematological observations on patients from our clinics (see [20, Table 1]). These distinctive features imply that individuals with HbSC disease should be treated as a discrete subset of SCD patients. 

Currently there is very little specific information on the pathophysiology and management of HbSC disease, with much being inferred from studies of HbSS patients. Differences in pathogenesis between HbSC and HbSS disease are expected, however. Understanding them will be important in the management of HbSC patients and may also contribute to a better appreciation of the condition in homozygous individuals.

## 4. Pathogenesis of SCD

Although the underlying molecular defect of SCD is long established, how HbS results in the clinical complications remains poorly understood. The chronic anaemia and acute ischaemic episodes both are associated with altered rheology and increased adhesiveness of both RBCs and vascular endothelium [21]. RBCs are more fragile and more readily scavenged from the circulation, contributing to the chronic anaemia, whilst microvascular occlusion is also encouraged causing the acute ischaemic events characteristic of SCD. Intravascular haemolysis is observed and the consequent release of Hb to circulate freely in plasma contributes to the vasculopathy, probably by scavenging nitric oxide (NO) and causing a functional deficiency of that molecule [22, 23]. Some authors divide the disease complications of SCD into two broad categories, with sequelae caused either predominantly by altered RBC rheology and elevated blood viscosity (e.g., pain, osteonecrosis, and acute chest syndrome) or by intravascular haemolysis and NO scavenging (e.g., pulmonary hypertension, stroke, priapism, and leg ulceration) [24–26]. In any event, polymerisation of HbS on deoxygenation is central to anaemia, vaso-occlusion, and haemolysis—although complete deoxygenation may not be needed, especially in the case of hyperdense RBCs with high cell [Hb], such as some of those found in HbSC individuals. Formation of long rods of HbS distorts RBC shape, reduces deformability, and increases viscosity, thus compromising vascular red cell rheology [27]. Other key events in the pathogenesis have been identified. First, red cell volume is critically important [28]. The increased cation permeability of HbS-containing red cells results in solute loss with water following osmotically. Consequently, [HbS] increases. As the rate of HbS polymerisation upon deoxygenation is proportional to a very high power of [HbS], a small reduction in cell volume and hence increase in [HbS] markedly encourages HbS polymerisation [27]. Second, red cell stickiness is also increased [21, 29–31]. This results, at least in part, from exposure of phosphatidylserine (PS) on the outer bilayer of the membrane [32]. Exposed PS is prothrombotic and increases adherence of red cells to macrophages and endothelium, contributing to chronic anaemia, haemolysis, and vaso-occlusion [33]. Again, the cause of PS exposure is not clear, but sickling-induced Ca^2+^ entry may play an important role [34]. Third, SCD represents an inflammatory state with raised levels of cytokines, chronic elevation of leukocyte counts, shortened leukocyte half-life, and abnormal activation of granulocytes, monocytes, and endothelium [35, 36]. The resulting cytokine stimulation of endothelial cells increases their adhesiveness to sickle RBCs [35, 36]. How these various changes interact to produce the symptoms of SCD represents a major research challenge. In addition, the extent to which these various mechanisms are involved in disease pathogenesis would be expected to differ between HbSS homozygotes and HbSC heterozygotes. For example, reduced intravascular haemolysis in the latter may ameliorate NO scavenging.

## 5. Altered Membrane Transport in Homozygous HbSS Cells

**Figure 1:**
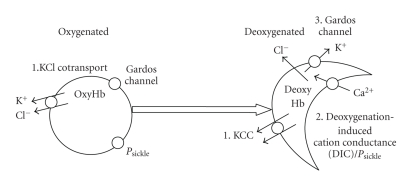
Schematic diagram of the main transport pathways activated in red blood cells (RBCs) from sickle cell patients. In RBCs from homozygous HbSS individuals, high cation permeability is accounted for by three main pathways [28, 37]. Under oxygenated conditions, the KCl cotransporter (KCC) is highly active. It is overexpressed in HbSS cells compared to HbAA ones and does not become quiescent as RBCs mature. It is stimulated further by low pH (reduction in extracelluar pH from 7.4 to 7). Under deoxygenated conditions, KCC remains active—again unlike the situation in HbAA RBCs [40]. In addition, two other pathways are observed. The deoxygenation-induced cation conductance (or *P*_sickle_) is activated as HbS polymerises. It mediates entry of Ca^2+^. Elevation in intracellular Ca^2+^ then leads to activation of the third pathway, the Ca^2+^-activated K^+^ channel, or Gardos channel. These three pathways result in solute loss, cell shrinkage and dehydration, and consequent increase in [HbS]. They thereby contribute to pathogenesis of sickle cell disease. They are also likely to be involved in solute loss from RBCs of patients heterozygous for HbS and HbC (HbSC genotype), though details are lacking and differences in their behaviour compared to that in HbSS cells are expected.

Increased membrane permeability of HbS-containing red cells contributes to SCD pathogenesis by promoting Ca^2+^ entry, KCl loss with water following osmotically, and hence RBC dehydration [28, 34, 37]. In HbSS cells, the involvement of three pathways has been proposed: the KCl cotransporter (KCC), the deoxygenation-induced cation conductance (or *P*_sickle_), and the Ca^2+^-activated K^+^ channel (or Gardos channel, KCNN4) [28]. These three systems are illustrated schematically in Figure 1. 

**Figure 2:**
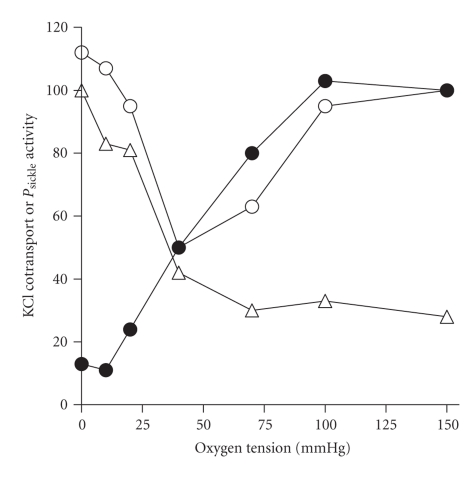
Effect of oxygen tension on the activity of KCl cotransport (KCC) or *P*_sickle_ in red blood cells (RBCs) from normal individuals or patients with sickle cell disease. The activity of each transport pathway is normalised—to the value in oxygenated cells (150 mmHg O_2_) for KCC activity and for that in deoxygenated RBCs (0 mmHg) in the case of *P*_sickle_—and given as a percentage. Solid circles give KCC activity in RBCs from normal HbAA individuals; open symbols give KCC activity (open circles) or *P*_sickle_ activity (open triangles) in RBCs from sickle cell patients (HbSS homozygotes). In these experiments, total magnitude of KCC activity was about 10-fold greater in RBCs from HbSS individuals compared with HbAA ones. Note how the deoxygenation-induced KCC activity and activation of *P*_sickle_ follow a similar dependence on O_2_ tension. Data taken from [67].

The first of these, KCC (likely KCC1 and KCC3 isoforms), is more active and abnormally regulated in HbSS cells [38–40]. Mean activity is enhanced >10-fold in unstimulated cells with several stimuli increasing activity further. In normal RBCs, cell swelling is an important trigger of KCC activity [41]. For HbSS cells, however, intracellular pH is probably the most important stimulus *in vivo*, with KCC activity reaching a peak at about pH 7 [38, 42]. The transporter also responds to O_2_ tension [43]. In normal red cells, high levels of O_2_ are required for KCC activity, with the transporter becoming inactivated at low O_2_. By contrast, in HbSS cells, the transporter remains active during full deoxygenation, thereby allowing it to respond to low pH in hypoxic areas (like active muscle beds) [40] (Figures 1 and 2). KCC is regulated by phosphorylation, through cascades of conjugate protein kinases and phosphatases [44], with differences apparent in HbSS cells compared with HbAA ones, but at present these are poorly defined. The relative deficiency of intracellular Mg^2+^ in HbSS cells [45, 46] probably acts to increase KCC activity by altering the activity of these regulatory enzymes.

**Figure 3:**
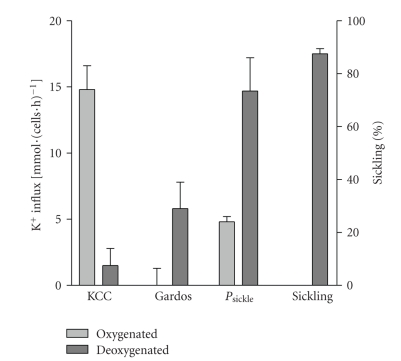
Components of K^+^ transport pathways and sickling in red blood cells (RBCs) from sickle cell disease patients heterozygous for HbS and HbC (HbSC genotype). K^+^ influxes are given as flux units [mmol·(l cells·h)^−1^] measured at 5 mM [K^+^]_o_ and numbers of sickled cells as a percentage of total RBCs in fully oxygenated (150 mmHg O_2_) or deoxygenated (0 mmHg) conditions. Although this technique measures a K^+^ influx, because of the high K^+^ content of RBCs, net solute movement through the transport systems will be outwards. KCl cotransport activity was calculated as the Cl^−^-dependent K^+^ influx, Gardos channel activity as the clotrimazole (5 *μ*M)-sensitive K^+^ influx, and *P*_sickle_ as the Cl^−^-independent K^+^ influx (Cl^−^ substituted with NO_3_^−^). Sickling, *P*_sickle_, and Gardos channel activation occurs in deoxygenated conditions—as for HbSS RBCs—but KCC activity is low when O_2_ is removed (as in RBCs from HbAA cells). Data taken from [64].

The second pathway, *P*_sickle_, is apparently unique to HbS-containing red cells [28, 34]. It is activated to a variable extent by deoxygenation, HbS polymerisation, and shape change [47, 48] (Figures 1 and 2). *P*_sickle_ has the characteristics of a nonspecific cation channel [34]. An anion permeability is controversial, whilst, more recently, it has been proposed as permeable under certain conditions to nonelectrolytes [49]. The main effect of *P*_sickle_ is probably the increased Ca^2+^ entry [49, 50] and possibly the Mg^2+^ loss [45]. Raised intracellular Ca^2+^ has several roles which include phospholipid scrambling [51]. It will also activate the third pathway responsible for HbS cell dehydration, the Gardos channel [52] (Figures 1 and 3). The Gardos channel is then capable of mediating very rapid efflux of K^+^ with Cl^−^ following for electroneutrality and water osmotically.

These mechanisms cause solute loss and HbSS cell shrinkage. Episodes may be short lived and produce only modest degrees of solute loss. But they may occur repeatedly during the lifetime of the RBCs, often during deoxygenation-induced sickling events. Accordingly, HbSS cells show an increase in MCHC of a few percent compared to normal red cells (c.34 g·dL^−1^ cf. 33 in HbAA cells, density approx 1.085 g·mL^−1^), but importantly there is a large range about this mean with many dense cells (>1.095 g·ml^−1^, MCHC c.38 g·dl^−1^), some of which are exceedingly dense (1.125 g·ml^−1^, c.50 g·dl^−1^) [53]. A significant feature of HbSS RBCs is their marked heterogeneity, with certain subpopulations possibly more important in pathogenesis [28]. The densest HbSS cells are mainly older ones, presumably following repeated episodes of solute loss [54]. Reticulocytes are mostly low density (c.26 g·dl^−1^), as they are in normal individuals [55]. However, there is a small fraction of young, dense HbSS cells, the so-called fast-track reticulocytes, which become rapidly dehydrated on deoxygenation while still young [28, 56].

## 6. Altered Properties of HbSC Cells

RBCs from HbSC patients also show K^+^ loss, raised MCHC and haemolytic anaemia with reticulocytosis [5, 57–59]. The properties of HbSC RBCs, however, differ in important respects from those of HbSS cells. In HbSC cells, K^+^ loss and dehydration are markedly more pronounced [58, 59]. MCHC is particularly high, at about 37 g·dl^−1^ (cf. 33 g·dl^−1^ in HbAA individuals; 33-34 g·dl^−1^ for the reversibly sickled fraction of HbSS patients) [57, 58]. Whilst most reticulocytes from normal HbAA and HbSS are characteristically low density (26 g·dl^−1^), HbSC reticulocytes are mainly high density (MCHC c.34 g·dl^−1^) [5, 58]. Usually older RBCs are denser; however the monotonic decrease in reticulocyte count with increasing cell density observed for red cells from HbSS patients (as well as HbAA and HbAS individuals) does not occur in HbSC patients [5, 60]. Instead, HbSC reticulocytes are fairly evenly distributed across the different RBC densities [5], or perhaps even more concentrated in the denser fractions [60]. This has been taken as evidence that a significant proportion of young HbSC cells begin their lives with a high density [5], rather than undergoing a more gradual dehydration observed in HbSS cells upon repeat episodes of sickling. In this respect, perhaps the majority of HbSC reticulocytes behave like the “fast-track” reticulocytes of HbSS patients [56]—cells which dehydrate rapidly on leaving the bone marrow—but this remains to be established. It also raises the question as to what constitutes RBCs in the less dense HbSC fractions. Can shrunken HbSC cells regain lost solute and increase their volume? If so, what is the mechanism and what are transport systems involved?

As heterozygotes, HbSC cells contain both HbS and HbC, in approximately equal amounts (i.e., 50%). This contrasts with the lower HbS content (c.40%) found in sickle trait HbAS cells [5]. Crystals of HbC are sometimes present in oxygenated RBCs. In contrast to HbS polymers, these deposits are lost on deoxygenation [60]. Because of the high HbS content and polymerisation, HbSC cells also show a deoxygenation-induced sickling shape change. In this case, however, rather than the HbSS sickles and holly leaf forms, deoxygenated HbSC cells show multifolded shapes such as “pita breads” and “tricorns” [60], perhaps because of the high surface area to volume ratio subsequent to their more marked dehydration. How HbS and HbC interact has also received some attention. Using different Hb mixtures, a direct interaction between the two Hbs appears to only slightly enhance HbS polymerisation. Much more important in HbSC disease is RBC dehydration and consequently the high MCHC [5, 61, 62]. High MCHC and lower levels of HbF may have an effect on the extent and kinetics of HbS polymerisation whilst concurrent Hb mutations (such as *β*-thalassaemia) may also play a significant role.

It is therefore critical to understand fully the mechanisms by which these RBCs shrink, but our understanding of the mechanisms involved remains uncertain. Oxygenated HbSC cells have elevated KCC activity that is stimulated by low pH and swelling [13, 60]. Cytoplasmic protein concentration has been suggested as the “volume” sensor of RBCs [63]. It is therefore intriguing to speculate that high KCC activity in oxygenated HbSC cells may result from the presence of HbC crystals which would lower the total concentration of soluble Hb, as occurs in swollen RBCs. In effect, the cells “think” that they are swollen and so activate mechanisms to lose solutes and water, namely, KCC. On the other hand, deoxygenated HbSC cells also show increased K^+^ efflux, to an extent apparently greater than that observed in deoxygenated HbSS cells [58]. Which pathway mediates the flux in deoxygenated conditions, however, has not been established. If *P*_sickle_ is involved, given the lower [HbS] of HbSC cells, it is not clear why it should be activated to a greater extent than in HbSS cells. KCC and the Gardos channel represent obvious alternative pathways.

A number of manoeuvres which reduce reticulocyte density may provide evidence for the transport pathways involved in their dehydration. Both Cl^−^ removal and deoxygenation shift HbSC reticulocytes to lower densities, consistent with solute retention following inhibition of KCC [60]. Hypotonic swelling of HbSC cells also reduces the deoxygenation-induced K^+^ loss [58], perhaps through reduction in [HbS] removing a *P*_sickle_-like element of K^+^ flux. 

**Figure 4:**
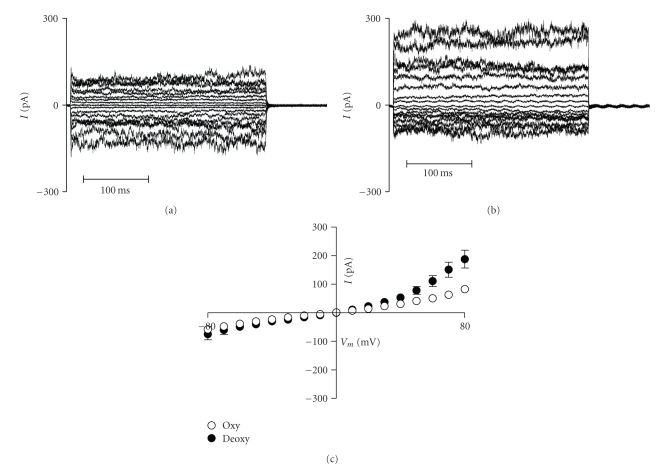
Conductance of red blood cells (RBCs) from sickle cell patients heterozygous for HbS and HbC (HbSC genotype). (a), (b) Representative whole-cell recordings from (a) oxygenated and (b) deoxygenated RBCs. (c) Mean whole-cell currents + S.E.M., *n* = 5. Test potentials from −80 to +80 mV were applied for 300 ms in 10 mV increments from a holding potential of −10 mV. Measurements were made using Na^+^-containing bath and pipette solutions. Data taken from [20]. See [66] for experimental details. The conductance of RBCs from HbSC patients is high and increases further on deoxygenation.

In preliminary studies, we have observed high KCC activity in oxygenated unfractionated HbSC cells, which was almost completely inhibited on deoxygenation [64]. Thus, KCC in HbSC cells behaved like that in HbSS cells at high O_2_ tension and like that in HbAA cells when tension was reduced [64] (Figure 3). We also found activation of a deoxygenation-induced Cl^−^-independent K^+^ flux [64], a deoxygenation-induced nonelectrolyte permeability [65] and a deoxygenation-induced rise in K^+^ conductance in patch-clamp experiments [66] (Figure 4), namely, a *P*_sickle_-like permeability, together with activation of the Gardos channel. In this context, it is interesting that HbC has a higher affinity for the RBC membrane than either HbA or HbS [59] leading to an early suggestion that HbSC interaction is involved in modulating RBC permeability. 

It is apparent, however, that our understanding of the permeability of HbSC cells requires further investigation.

## 7. Conclusion: The Importance of Cell Dehydration in HbSC Disease

Understanding dehydration is particularly relevant for HbSC cells. The solubility of deoxygenated HbS is about 17 g·dl^−1^ compared to 70 g·dl^−1^ for HbA. As HbS represents only about half the total Hbs in HbSC cells, a relatively small decrease in MCHC (from an RBC total of 37 to 33 g·dl^−1^) will prevent HbS polymerisation [61] while retaining the functionally important discocyte morphology. As HbS constitutes about half the Hbs in these RBCs, this would mean a fall in [HbS] from 18.5 to 16.5. In comparison, in HbSS cells, a reduction of MCHC to <25 g·dl^−1^ is required, by which time RBCs will be spherocytic, and cell swelling *per se *will adversely affect rheology [58]. Notwithstanding their relevance to dehydration and sickling, the permeability of HbSC cells has not been well studied nor compared in detail with that of HbSS cells. Several areas require more careful investigation. The interaction between different Hbs, membrane target sites regulating permeability, the transport pathways involved, the role of cell density, oxygenation, volume, and pH presents a complex pattern of modalities controlling solute content and hence cell density and MCHC. Control by phosphorylation remains mainly unexplored. The challenge ahead lies to define the most important stimuli and how they interact to determine cell volume. A major therapeutic goal is the ability to prevent HbSC cell dehydration or to promote rehydration.
